# Transmission dynamics of drug-resistant tuberculosis in Ningbo, China: an epidemiological and genomic analysis

**DOI:** 10.3389/fcimb.2024.1327477

**Published:** 2024-02-07

**Authors:** Yang Che, Xiangchen Li, Tong Chen, Yewei Lu, Guoxin Sang, Junli Gao, Junshun Gao, Zhengwei Liu, Tianfeng He, Yi Chen

**Affiliations:** ^1^ Institute of Tuberculosis Prevention and Control, Ningbo Municipal Center for Disease Control and Prevention, Ningbo, Zhejiang, China; ^2^ Key Laboratory of Precision Medicine in Diagnosis and Monitoring Research of Zhejiang Province, Hangzhou, Zhejiang, China; ^3^ The Institute of Tuberculosis (TB) Control, Zhejiang Provincial Center for Disease Control and Prevention, Hangzhou, Zhejiang, China

**Keywords:** tuberculosis, drug resistance, molecular epidemiology, recent transmission, spatial distribution, whole-genome sequencing, phylodynamics

## Abstract

**Background:**

Tuberculosis (TB), particularly drug-resistant TB (DR-TB), remains a significant public health concern in Ningbo, China. Understanding its molecular epidemiology and spatial distribution is paramount for effective control.

**Methods:**

From December 24, 2020, to March 12, 2023, we collected clinical *Mycobacterium tuberculosis* (MTB) strains in Ningbo, with whole-genome sequencing performed on 130 MTB strains. We analyzed DR-related gene mutations, conducted phylogenetic and phylodynamic analyses, identified recent transmission clusters, and assessed spatial distribution.

**Results:**

Among 130 DR-TB cases, 41% were MDR-TB, 36% pre-XDR-TB, 19% RR-TB, and 3% HR-TB. The phylogenetic tree showed that 90% of strains were Lineage 2 (Beijing genotype), while remaining 10% were Lineage 4 (Euro-American genotype). The spatial analysis identified hotspots of DR-TB in Ningbo’s northern region, particularly in traditional urban centers. 31 (24%) of the DR-TB cases were grouped into 7 recent transmission clusters with a large outbreak cluster containing 15 pre-XDR-TB patients. Epidemiological analyses suggested a higher risk of recent DR-TB transmission among young adult patients who frequently visited Internet cafes, game rooms, and factories.

**Conclusion:**

Our study provides comprehensive insights into the epidemiology and genetics of DR-TB in Ningbo. The presence of genomic clusters highlights recent transmission events, indicating the need for targeted interventions. These findings are vital for informing TB control strategies in Ningbo and similar settings.

## Introduction

Tuberculosis (TB) is an infectious disease caused by *Mycobacterium tuberculosis* which seriously damages human health, especially among some low- and middle-income populations. In 2021, 10.6 million new cases of TB were diagnosed globally, with an incidence rate of 134 per 100,000 and 1.6 million deaths from TB (Bagcchi, 2023). The World Health Organization (WHO) proposed a strategy to eliminate TB, which was to reduce TB mortality to less than 95% and incidence to 90% by 2035 (Bagcchi, 2023). However, the emergence of drug-resistant TB (DR-TB) has seriously hindered the prevention and treatment of TB. DR-TB is now becoming the world’s deadliest pathogen, with a quarter of deaths attributed to antimicrobial drug resistance (Reid et al., 2019). Close to half a million people developed rifampicin-resistant TB (RR-TB), of which 78% had multidrug-resistant TB (MDR-TB) in 2019 (WHO, 2020). Resistance to anti-TB drugs often means fewer treatments, poorer outcomes, and higher medical costs.

The movement of populations that exacerbates the global epidemic can lead to the transmission of DR-TB extending within individual cities and even across national borders (Walker et al., 2018; Bainomugisa et al., 2019). China’s urbanization process in recent decades has been characterized by the rapid expansion of urban centers, largely attributed to the migration of young individuals from rural regions enticed by employment prospects (Yang et al., 2021). This substantial influx of migrants to cities has posed significant challenges to the control and prevention of TB. WHO has listed China as a high-burden country for TB, TB and HIV co-infection, and multidrug-resistant TB (MDR-TB) during the period 2016–2020 (Bagcchi, 2023). Recent transmission is primarily responsible for driving the global endemic of MDR-TB (Kendall et al., 2015; Yang et al., 2017). Therefore, comprehending the transmission patterns of MDR-TB is essential for guiding effective public health interventions.

Ningbo is a major sub-provincial city in northeast Zhejiang Province, China. With a population of about 9.4 million, Ningbo was established as the most special economic zone in eastern China and a major port for foreign trade. The 7th National Census in 2020 reported that Ningbo city has attracted 4.5 million migrants from different regions in China. Although the overall incidence of TB in Ningbo is low in comparison to other regions in China, the immigrant populations in Ningbo show relatively a high prevalence of DR-TB, which highlights the need for clinical control of tuberculosis (Jiang et al., 2021; Zhou et al., 2022). Despite its importance, the intricacies of DR-TB transmission in Ningbo remain underexplored.

This study aims to unravel the complex dynamics of DR-TB transmission in Ningbo, China, employing a comprehensive approach that melds epidemiological, molecular genetics, and spatial analytical methods. Our objectives encompass deciphering recent transmission patterns of DR-TB, identifying pivotal risk factors, and pinpointing geographical areas of heightened risk within Ningbo. Our findings establish a scientific foundation, enabling public health agencies to formulate more effective strategies for the control and prevention of tuberculosis.

## Methods

### Isolate collection and genomic DNA extraction

The study encompassed patients with pulmonary disease and culture-positive MTB sampled during DR-TB surveillances in Ningbo City, Zhejiang Province, Eastern China. Genomic DNA from MTB colonies scraped from the L-J medium was detected to obtain for sequencing by the CTAB technique of DNA purification. Each extracted DNA was quantified by Qubit 2.0 Fluorometer (Invitrogen, Carlsbad, CA, USA).

### Drug susceptibility test

Drug susceptibility tests of four first-line anti-TB drugs and five second-line drugs were carried out based on WHO recommendations (World Health Organization, 2020). The drug concentrations are isoniazid (INH) 0.2 µg/ml, rifampicin (RIF) 40 µg/ml, ethambutol (EMB) 2 µg/ml, streptomycin (SM) 4 µg/ml, levofloxacin (LVX) 2 µg/ml, amikacin (AMK) 30 µg/ml, capreomycin (CM) 40 µg/ml, ethionamide (ETO) 40 µg/ml, and para-aminosalicylic acid (PAS) 1 µg/ml. H37RV strains were used as a reference for quality control. According to the definition formulated by WHO, DR-TB encompasses various types, including isoniazid-resistant (HR)TB, rifampicin-resistant (RR)-TB, and multi-drug resistant (MDR)-TB (resistant to both rifampicin and isoniazid). Additionally, it includes pre-extensively drug-resistant TB (pre-XDR-TB), characterized by resistance to rifampicin (MDR/RR-TB), and any fluoroquinolone. (World Health Organization, 2020).

### WGS and bioinformatics

The whole genomic DNA was used to prepare 150 bp pair-end libraries and sequenced on an Illumina NovaSeq 6000 platform with 150 cycles, aiming for a coverage depth of 200×. To ensure specificity to MTB, we employed Kraken v1.1.1 with the prebuilt MiniKraken DB_8GB database, accepting only those isolates with a minimum of 90% of reads mapping to *Mycobacterium tuberculosis* complex (Wood and Salzberg, 2014; Vargas et al., 2021). The quality and integrity of the accepted FASTQ files were assessed, and low-quality regions were trimmed using fastp v0.23, ensuring an average read quality of Q20 (Chen et al., 2018).

Genetic variations were identified by aligning the filtered reads to the reference genome H37Rv (GenBank: NC000962.3) using BWA-MEM v0.7.17 with default parameters (Li, 2013). Subsequently, the mapped reads underwent processing using SAMtools v1.9 and the Genome Analysis Toolkit (GATK) v4.0.5 (Li et al., 2009; McKenna et al., 2010). This included base recalibration and realignment to address potential artifacts. High-confidence single nucleotide polymorphisms (SNPs) were identified using the SAMtools/BCFtools suite, considering only loci with fixed alternate alleles (frequency ≥ 90%) supported by at least five reads from both forward and reverse sequencing (Liu et al., 2020). MTB lineages and drug resistance-associated mutations were determined using TB-Profiler v4.4.2 (Phelan et al., 2019).

### Phylogenetic and phylodynamic analysis

Fixed SNPs, excluding those within the proline-glutamic acid-proline-proline-glutamic acid sequence, the proline-glutamic acid-polymorphic GC-rich sequence, and drug resistance-associated genes, were consolidated into a concatenated alignment. (Luo et al., 2014). Maximum-likelihood (ML) phylogenetic trees were constructed from this concatenated alignment using IQ-Tree v2.2.2 for all MTB isolates (Nguyen et al., 2015). The tree construction employed the following parameters: “-m TEST -B 1000”, signifying automatic model selection by jModelTest, along with 1000 ultrafast bootstrap replicates (Darriba et al., 2012). The best-scoring ML tree was rooted using *Mycobacterium canettii* (RefSeq: NC_015848.1) as the outgroup and was visualized using the Interactive Tree of Life (iTOL) (Letunic and Bork, 2016).

### Spatial and statistical analysis

The residential addresses of TB patients at the time of diagnosis were geocoded using a Chinese-language-based web geocoding tool provided by Baidu Maps (Baidu, Beijing, China). Geographical distances between these geocoded locations were calculated using the distHaversine function from the R package geosphere (Hijmans et al., 2017). Two-dimensional kernel density estimation was conducted with the R package MASS to identify spatial aggregation patterns among DR-TB patients (Ripley et al., 2013). Statistical analyses were performed using the R package gtsummary (Sjoberg et al., 2021). Associations with genomic clustering were assessed using the chi-square test. In cases where the sample size was insufficient (i.e., more than 20% of cells had expected frequencies < 5), Fisher’s exact test was employed. Variables with a P-value below 0.05 were deemed statistically significant in their association with genomic clustering. The results of spatial and statistical analyses were visualized using the R packages ggplot2 and ggpubr (Wickham, 2016; Kassambara, 2020).

### Interview and social network analysis

Patients with strains belonging to genomic clusters comprising at least three cases were invited to partake in structured, in-depth interviews regarding their social networks. Following informed consent, enrolled patients completed a questionnaire to provide data on close contacts, social interactions, and frequented locations over the five years preceding their TB diagnosis (Yang et al., 2017). This information comprised detailed addresses of their homes, workplaces, and public community centers and facilities.

## Results

### Basic description of cases of DR-TB in Ningbo

**Figure 1:**
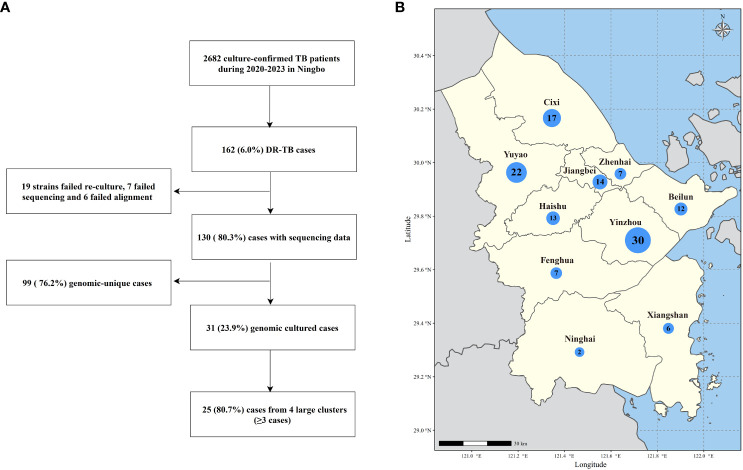
**(A)** Sample enrollment and study flowchart. **(B)** Map of Ningbo City showing the number of sequenced DR-TB isolates from each county and district under Ningbo. The size of the bubbles is proportional to the number of isolates.

From December 24, 2020, to March 12, 2023, the Ningbo CDC collected and stored 2682 clinical MTB strains, and 162 (6.0%) were DR-TB. WGS was successfully performed on strains isolated from 130 patients of DR-TB (**Figure 1A**, **Supplementary Table S1**). Among the 130 sequenced DR-TB cases, various resistance patterns were identified by DST, with 53 cases (40.8%) characterized as MDR-TB, 47 cases as pre-XDR-TB (36.2%), 24 cases (18.5%) as RR-TB, 4 cases (3.1%) as HR-TB, and 2 cases (1.5%) only resistant to levofloxacin. Among the MDR-TB cases, 46.5% (46/99) had additional resistance to fluoroquinolones (so-called pre-XDR).

The median age of the patients was 45 years (interquartile range, 31-58 years). Among these patients, residents and migrants accounted for 53.1% (69/130) and 46.9% (61/130), respectively. 78.5% (102/130) of the patients were male, and 46.9% (61/130) were treatment-naïve. 24.6% (32/130) and 33.8% (44/130) of the patients were farmers and factory workers, respectively. Importantly, DR-TB patients are found in all seven counties and cities under Ningbo (**Figure 1B**), with the highest number in Yinzhou District (n=30) and the lowest number in Ninghai County (n=2).

To explore factors associated with elevated levels of drug resistance, we examined the correlations between MDR-TB and pre-XDR-TB with bacteriological, demographic, clinical, and geographic factors. However, no statistically significant associations were identified. (**Supplementary Table S2**).

### Drug-resistance-related gene mutations

Genetic drug-resistance profiling was conducted through WGS data. 37.7% (49/130) of the DR-TB strains exhibited drug-resistant mutations in *gyrA*/*B*, consequently falling into the categories of fluoroquinolone-resistant and pre-XDR-TB strains. Notably, none of these strains were identified as XDR-TB. The most recurrent mutations encompassed Ser450Leu (71/130) in *rpoB* (RIF), Ser315Thr (61/130) in *katG* (INH), Met306Val (30/130) in *embB* (EMB), Val139Leu (15/130) in *pncA* (PZA), Lys43Arg (62/130) in *rpsL* (SM), and Asp94Gly (31/130) in *gyrA* (FQ).

Furthermore, we assessed the predictive capability of genotypic DST based on WGS (**Supplementary Table S3**). The analysis demonstrated an average concordance rate of 92.4% (130 strains) across all nine drugs, with values ranging from 77.7% (EMB) to 98.5% (AMK). The collective range of sensitivity and specificity for WGS were 44.4% (PAS) to 100% (RIF), and 68.2% (EMB) to 100.00% (AMK), respectively. The phenotypic DST indicated drug resistance in 26 samples (10 for PAS, 4 for INH, 2 for SM, EMB, AMK, CM, LVX, and ETO); however, the WGS results did not reveal any known drug-resistant mutations in these samples.

### Phylogenetic analysis of DR-MTB strains in Ningbo

**Figure 2:**
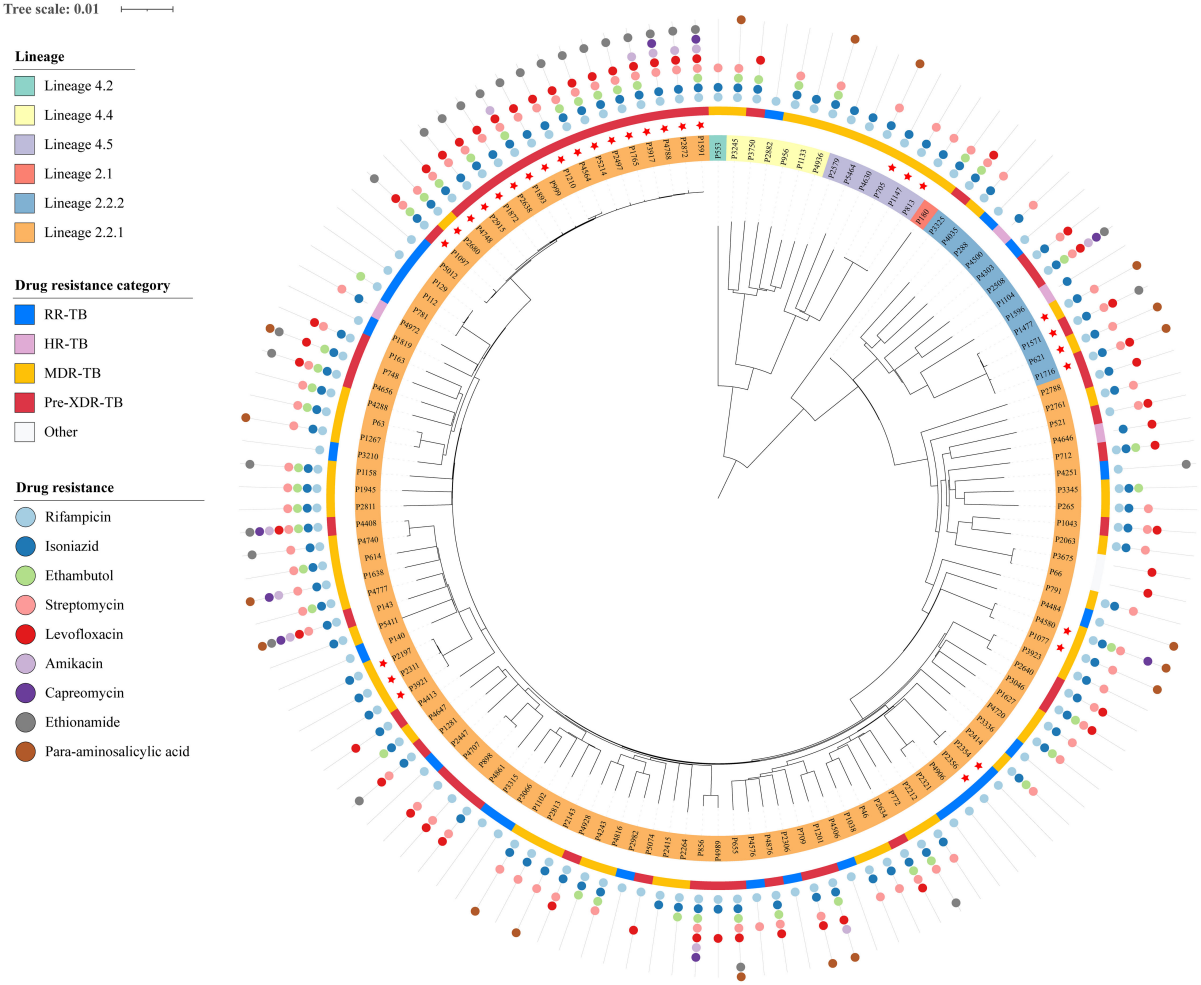
Phylogeny and resistance profile of 130 DR-MTB strains. Tip nodes were colored according to the lineages and sub-lineages determined by the TB profiler. The outer yellow-orange-red circle shows the types of DR-TB. The outermost colored dots indicate the resistance to nine anti-TB drugs.

To elucidate the evolutionary relationships of these strains, we reconstructed an ML phylogenetic tree using concatenated sequences derived from non-redundant SNP loci of all 130 DR-TB strains in Ningbo (**Figure 2**). Genotyping analysis unveiled that 90.0% (117/130) of the strains belonged to lineage 2 (L2), with the majority falling under L2.2.1 and L2.2.2 (Beijing family strains), accounting for a total of 116 cases. Only one strain was classified as L2.1. The remaining 10.0% (13/130) of the strains belonged to lineage 4 (L4), comprising L4.4 (n=6), L4.5 (n=6), and L4.2 (n=1), respectively.

In addition, to assess whether the genotypes of strains in Ningbo differed from those isolated in other areas, we constructed an ML phylogenetic tree using our strains in combination with those collected from various regions across China (**Supplementary Figure S1**, **Supplementary Table S4**). The phylogenetic analysis revealed that most of the strains in Ningbo were mixed with those from other regions with deep branches. However, there were 13 sub-lineages containing 48 strains exclusively from Ningbo with very small genetic distances, and 93.8% (45/48) were Beijing family strains.

### Identification of genomic-clustered DR-TB cases and their risk factors

Transmission clusters were characterized by strains differing by a maximum of 12 SNPs (Yang et al., 2017). A total of 31 strains were grouped into 7 genomic clusters (**Supplementary Table S5**), constituting a clustering rate of 23.8% (31/130). Notably, the largest cluster (designated as cluster 1) encompassed 15 strains, while the remaining clusters (clusters 2-6) contained between 2 and 4 strains. Additionally, it was worth mentioning that out of these clusters, 4 contained a mix of resident and migrant patients, while the remaining 2 clusters consisted exclusively of either resident or migrant patients.

To comprehensively evaluate factors linked to genomic clustering, we examined the correlations between clusters and bacteriologic, demographic, clinical, and geographic variables. (**Table 1**). An age distribution analysis revealed significance (Fisher’s exact test, *P* = 0.005), uncovering varied age group proportions. Notably, the 18-37 age group demonstrated a heightened transmission risk.

**Table 1:** Characteristics of genomic-clustered and unique cases of DR-TB in Ningbo, China.

	No. (%)			χ^2^	*P* value
	Clustered (n = 31)	Unique (n = 99)	Total (n = 130)		
Bacteriological factors					
** Genotype**					>0.9*^2^*
** **Beijing	28 (90%)	89 (90%)	117 (90%)		
** **Non-Beijing	3 (9.7%)	10 (10%)	13 (10%)		
** Drug resistance category**					0.064*^2^*
** **RR-TB	3 (9.7%)	22 (22%)	24 (19%)		
** **HR-TB	0 (0%)	4 (4.0%)	4 (3.1%)		
** **MDR-TB	11 (35%)	42 (42%)	53 (41%)		
** **Pre-XDR-TB	17 (55%)	29 (29%)	47 (35%)		
** **Other	0 (0%)	2 (2.0%)	2 (1.5%)		
Demographic factors					
** Gender**				0.70	0.4*^1^*
** **Female	5 (16%)	23 (23%)	28 (22%)		
** **Male	26 (84%)	76 (77%)	102 (78%)		
** Age**					**0.005** *^2^*
** **18-27	7 (23%)	16 (16%)	23 (18%)		
** **28-37	12 (39%)	15 (15%)	27 (21%)		
** **38-47	2 (6.5%)	15 (15%)	17 (13%)		
** **48-57	1 (3.2%)	25 (25%)	26 (20%)		
** **58-	9 (29%)	28 (28%)	37 (28%)		
** Occupation**				3.0	0.2*^1^*
** **Factory/Office worker	16 (52%)	43 (43%)	59 (45%)		
** **Farmer	4 (13%)	28 (28%)	32 (25%)		
** **Unemployed	11 (35%)	28 (28%)	39 (30%)		
Clinical factors					
** TB history**				0.05	0.8*^1^*
** **Yes	17 (55%)	52 (53%)	69 (53%)		
** **No	14 (45%)	47 (47%)	61 (47%)		
** Treatment**				0.05	0.8*^1^*
** **New case	14 (45%)	47 (47%)	61 (47%)		
** **Retreated case	17 (55%)	52 (53%)	69 (53%)		
** Smear**				0.04	0.8*^1^*
** **+	16 (52%)	49 (49%)	65 (50%)		
** **-	15 (48%)	50 (51%)	65 (50%)		
Geographic factors					
** Household**				0.41	0.5*^1^*
** **Resident	18 (58%)	51 (52%)	69 (53%)		
** **Migrant	13 (42%)	48 (48%)	61 (47%)		

^1^Pearson’s Chi-squared test; ^2^Fisher’s exact test

### Genetic and epidemiological patterns of genomic-clustered cases

The genomic clusters could signify the transmission of a DR-TB strain or the initial transmission of a non-DR-TB strain that subsequently developed multidrug resistance. Upon comparing drug-resistance mutation profiles, we found consistent mutations associated with isoniazid and rifampicin resistance in all seven genomic clusters, confirming the transmission of DR-TB strains rather than acquired resistance. (**Supplementary Table S5**). Furthermore, many other mutations associated with drug resistance were also consistent within all of the strains in these 7 clusters. Taken together, all genomically clustered cases of DR-TB cases resulted from recent transmission.

**Figure 3:**
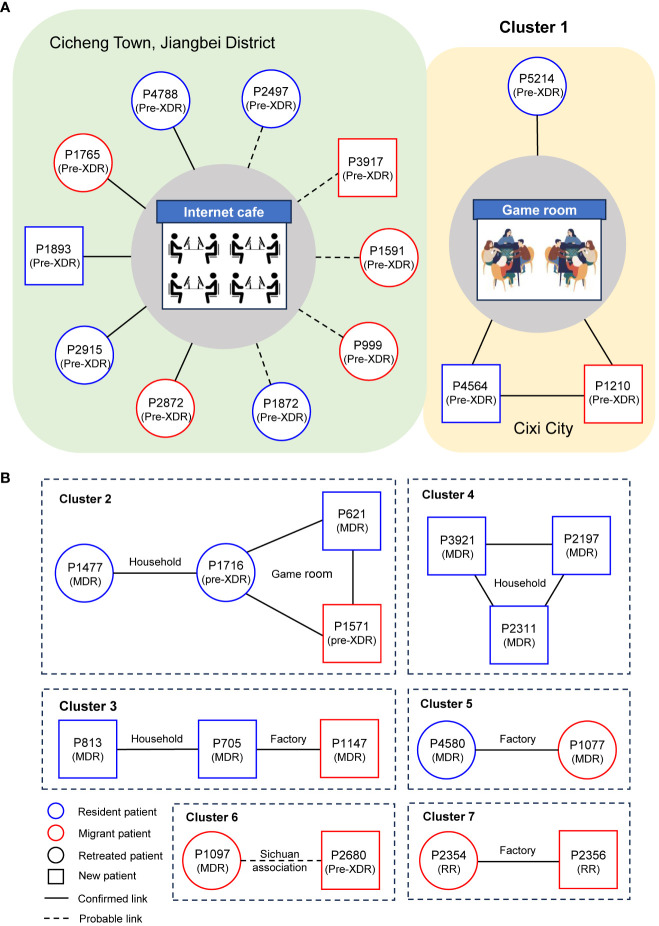
Results of the detailed epidemiological investigation of the largest cluster **(A)** and other clusters **(B)**. Each circle represents a DR-TB patient. The text in brackets below the case number in the node indicates the type of resistance.

To elucidate transmission links among genomic-clustered cases, in-depth interviews of social networks were conducted for all seven genomic clusters (**Figure 3**). For the largest cluster (cluster 1), we successfully obtained epidemiological data for 11 patients. All patients in cluster 1 were male, with ages ranging from 25 to 73 years and a median age of 29 years. Five patients were known to have used an Internet cafe in Cicheng Town, Jiangbei District, between 2016 and 2020, while an additional five patients had documented contact with other DR-TB patients associated with other Internet cafes in Cicheng. Notably, eight (80%) of the ten patients in the internet cafes were retreated ones. Moreover, two patients had shared exposure at a game room in Kandun Street, Cixi City, while one patient had a history of extended visits to multiple game rooms in Cixi before falling ill. However, the epidemiological link between exposed patients in Internet cafes and game rooms was unclear.

Follow the above strategy, confirmed or probable epidemiological links were successfully identified in 29 (94%) out of 31 patients. Among the established epidemiological links, we found 1 cluster included 10 patients who shared exposure to Internet cafes, 3 clusters included 7 family members, 3 clusters included 6 workers from the factories, and 2 clusters included 6 patients who attended the game rooms. Patients in cluster 6 were probably linked because they came from the same county in Sichuan Province. Additionally, 5 (71%) of the 7 genomic clusters suggested that transmission of DR-TB between residents and migrants occurred.

### Spatial analysis of DR-TB cases

**Figure 4:**
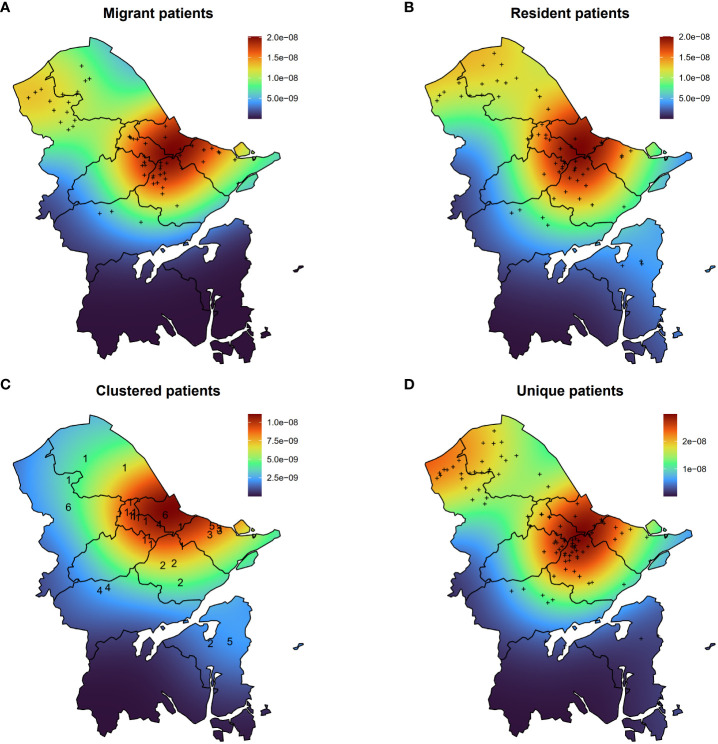
Kernel density maps of migrant patients **(A)**, resident patients **(B)**, clustered patients **(C)**, and unique patients **(D)**. The numbers in **(C)** indicate the number of the genomic cluster to which the case belongs.

To gain a comprehensive insight into spatial distribution, kernel density maps were generated using the residential addresses of both migrant and resident DR-TB patients. The concentration of DR-TB cases in Ningbo was predominantly observed in the northern region (**Figures 4A, B**). Notably, the spatial hotspots for both migrant and resident patients coincided, concentrating at the intersection of the primary urban areas, namely Haishu, Jiangbei, Zhenhai, Yinzhou, and Beilun Districts. These districts constitute the traditional urban center of Ningbo, characterized by high population density.

Furthermore, an analysis of clustered and unique patients unveiled varying geographical distributions (**Figures 4C, D**). The hotspot for clustered patients was identified in Jiangbei District, while two hotspots were observed for unique patients. One was in the adjacent area of Haishu and Yinzhou Districts, and the other encompassed the northern region of Yuyao City.

Upon examining the living addresses within each genomic cluster, we determined that the geographical distance among clustered cases ranged from 0 to 58.9 kilometers (Km), with an average paired distance of 39.2 Km (interquartile range, 19.6–55.7 Km). Notably, four out of seven clustered cases originated from the same area, while the remaining three hailed from distinct locales (**Figure 4C**). By setting the same community as residing within a two-kilometer radius, patients from two out of six clusters could be identified as living in the same community, with only the patients in cluster 3 residing within the same district. Additionally, we observed a higher positive correlation (*R*=0.36, *P*=4.9e-5) between the geographical distance among clustered patients and the SNP distance between MTB strains compared to the correlation observed among unique patients (*R*=0.083, *P*=8.7e-12) (**Supplementary Figure S2**).

## Discussion

In this investigation, we integrated WGS, spatial analysis, and epidemiological research to delineate the transmission patterns of DR-TB over a 3-year period in Ningbo, a major metropolis on the East Coast of China. We found that more than three-quarters (76.9%) of all DR-TB cases were MDR-TB or pre-XDR-TB. The DR-TB cases were concentrated in the northern region of Ningbo, particularly in the traditional urban center, which is characterized by high population density. Based on the genomic clustering, we successfully retrieved epidemiological links among residential communities or complexes, and related public facilities, such as Internet cafes, game rooms, and factories.

In Ningbo, strains from various local sub-lineages within the L2 Beijing family branch were identified exclusively through the reconstruction of a phylogenetic tree that included strains from other regions of China. The Beijing family strains have prominently characterized the MTB population in China for the past century (Liu et al., 2018). Recent transmission accounted for approximately a quarter (24%) of all DR-TB cases in our study. This proportion, while higher than that reported in low-burden countries such as the USA and the UK (Moonan et al., 2013; Anderson et al., 2014), showed little difference from developed regions in China, such as Beijing (25%), Shanghai (32%), and Shenzhen (25%) (Yang et al., 2017; Jiang et al., 2020; Yin et al., 2022). In a multi-setting study within China, 43% of MDR-TB cases were found in genotypic clusters, and DR itself emerged as an independent risk factor for the recent transmission of MTB strains. Moreover, modeling studies have indicated a troubling trend of unfavorable TB treatment outcomes, potentially associated with the rising prevalence of MDR-TB and the comparatively low rate of MDR detection and treatment in China. These findings collectively underscore the possibility of MDR-TB transmission being more extensive than observed in our present study.

We did not find specific populations in Ningbo at an elevated risk of DR-TB transmission, except for young adults in the 18-37 age group. The identification of epidemiological links highlighted a high risk for DR-TB outbreaks in unregulated Internet cafes and both young residents and migrants who spend most of their time there. A recent study in Shanghai also reported an outbreak of TB in Internet cafes among other young internal migrants without fixed abode (Lu et al., 2023). This high-risk population shares characteristics such as unstable housing, overcrowded environments like Internet cafes and factories, malnutrition, and challenges in healthcare system identification, all contributing to their elevated TB transmission risk (Lu et al., 2023). In addition, although TB treatment history is not related to transmission, we found that most of these cases in Internet cafes were retreated TB patients. Accordingly, targeted interventions aimed at this population and strengthening supervision and education on Internet cafes and game rooms could be strategic approaches in controlling the spread of DR-TB.

Transmission of DR-TB in Ningbo appeared to be in the traditional city center of Ningbo with high population density. We noted a significant correlation between the proximity of residences among DR-TB patients and the genetic similarity of the MTB strains causing their infections. In a prior study in Shanghai, it was observed that with each additional kilometer between the residences of two patients, the likelihood of genomic clustering (defined as sharing 10 or fewer SNPs) decreased by 10%. (Yang et al., 2018). These findings imply that in developed cities in China, TB patients in close geographical proximity are more likely to belong to a genomic cluster, underscoring the potential benefits of conducting contact investigations.

The majority of drugs based on WGS showed sensitivity and specificity exceeding 90%, affirming the reliability of WGS as a method for predicting drug resistance. However, our study revealed a lower specificity in predicting EMB resistance compared to other drugs. The differences between WGS and phenotypic DST arise from several factors. Firstly, phenotypic DST may overlook samples with low drug resistance. Notably, it has limitations in detecting EMB resistance, particularly in cases of INH resistance (Meehan et al., 2019). Nevertheless, the impact of resistance mechanisms to other drugs on EMB resistance remains unclear (Heyckendorf et al., 2018). Secondly, WGS may be limited in diagnosing specific types of non-specific resistance, such as resistance caused by efflux pumps (Kanji et al., 2019).

Several limitations should be acknowledged. Firstly, our ability to infer DR-TB transmission patterns in Ningbo relied on the completeness of our MTB isolate sampling, which was not exhaustive due to non-culture-positive registered cases and the inclusion of only 80% of DR-TB patients in Ningbo. Secondly, the retrospective study design and limited timeframe may not have fully represented all DR-TB cases within the TB population. Thirdly, we might have overlooked cases among migrants who sought treatment in their home province, potentially resulting in an underestimated contribution of migrants to disease transmission.

In conclusion, our study provides valuable insights into the transmission patterns and risk factors associated with DR-TB in Ningbo. The identification of genomic clusters, drug resistance variations, age-related transmission risks and public facilities, and geographical disparities underscores the complexity of DR-TB transmission. Tailored interventions, increased surveillance, and further research are needed to address these challenges effectively and reduce the burden of DR-TB in Ningbo and beyond.

## Data availability statement

The original contributions presented in the study are publicly available. This data can be found here: https://ngdc.cncb.ac.cn/bioproject/browse/PRJCA018599.

## Ethics statement

The studies involving humans were approved by Ethics Committees of Ningbo Municipal Center for Disease Control and Prevention. The studies were conducted in accordance with the local legislation and institutional requirements. Written informed consent for participation in this study was provided by the participants’ legal guardians/next of kin. Written informed consent was obtained from the individual(s), and minor(s)’ legal guardian/next of kin, for the publication of any potentially identifiable images or data included in this article.

## Author contributions

YaC: Formal Analysis, Visualization, Writing – original draft, Writing – review & editing. XL: Formal Analysis, Writing – original draft, Writing – review & editing. TC: Data curation, Writing – review & editing. YL: Formal Analysis, Writing – review & editing. GS: Conceptualization, Data curation, Formal Analysis, Writing – review & editing. JLG: Project administration, Writing – review & editing. JSG: Project administration, Resources, Writing – review & editing. ZL: Funding acquisition, Supervision, Writing – review & editing. TH: Funding acquisition, Supervision, Writing – review & editing. YiC: Funding acquisition, Supervision, Writing – review & editing.
