# Supplementary material for: Transmission dynamics of drug-resistant tuberculosis in Ningbo, China: an epidemiological and genomic analysis
Source: Front Cell Infect Microbiol. 2024 Feb 7;14:1327477. doi: 10.3389/fcimb.2024.1327477 (PMC10879548; doi:10.3389/fcimb.2024.1327477)
Supplement: Supplementary file 1 [file Image_1.pdf]

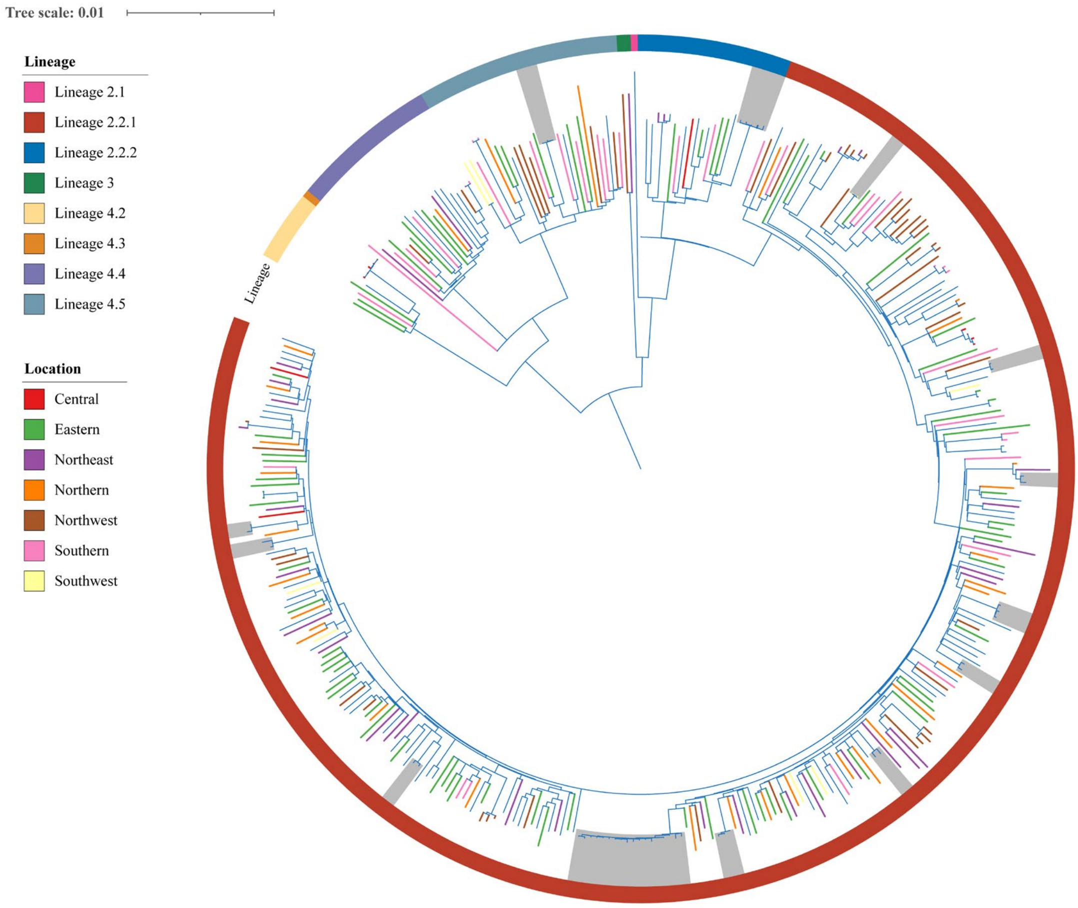

Supplemental Figure S1. Phylogenetic tree of 130 DR-TB strains found in Ningbo and other 246 TB strains detected in other Chinese area. Strains with blue lines were collected in Ningbo, while those with other ones were obtained in other parts of China as previously published. The grey areas indicate the sub-lineages exclusively from Ningbo.
