# Supplementary material for: Transmission dynamics of drug-resistant tuberculosis in Ningbo, China: an epidemiological and genomic analysis
Source: Front Cell Infect Microbiol. 2024 Feb 7;14:1327477. doi: 10.3389/fcimb.2024.1327477 (PMC10879548; doi:10.3389/fcimb.2024.1327477)
Supplement: Supplementary file 2 [file Image_2.pdf]

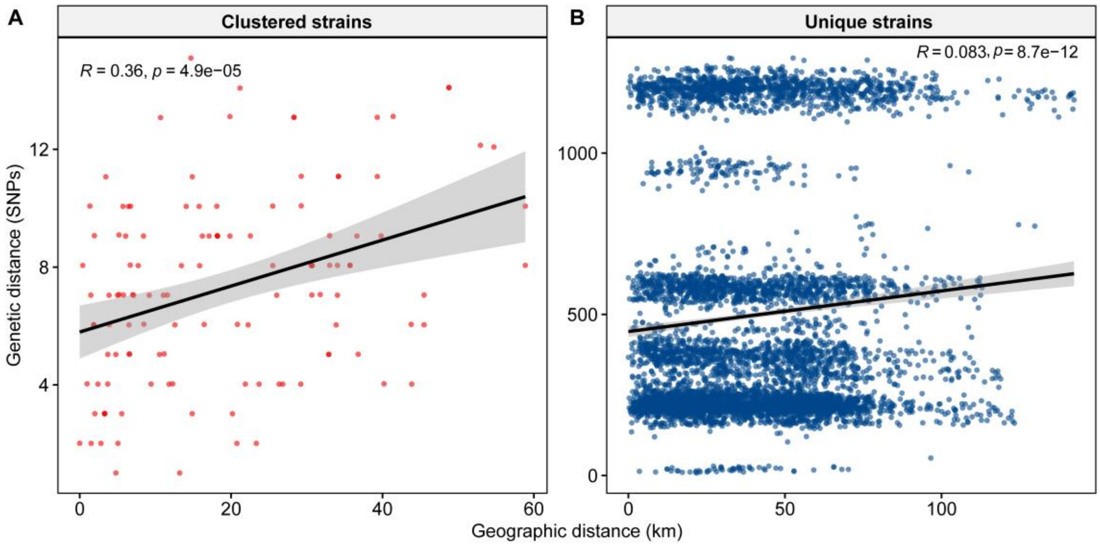


**Supplementary Figure S2**. Correlation between pairwise SNP distance and pairwise geographic distance for genomic clustered groups ≤12 SNPs (A) and unclustered cases (B). SNP, single-nucleotide polymorphism.
