# Supplementary figures and images for: Transmission dynamics of drug-resistant tuberculosis in Ningbo, China: an epidemiological and genomic analysis
Source: Front Cell Infect Microbiol. 2024 Feb 7;14:1327477. doi: 10.3389/fcimb.2024.1327477 (PMC10879548; doi:10.3389/fcimb.2024.1327477)

**Supplemental Table S2. Characteristics of MDR/Pre-XDR-TB and Other DR-TB patients in Ningbo, China**


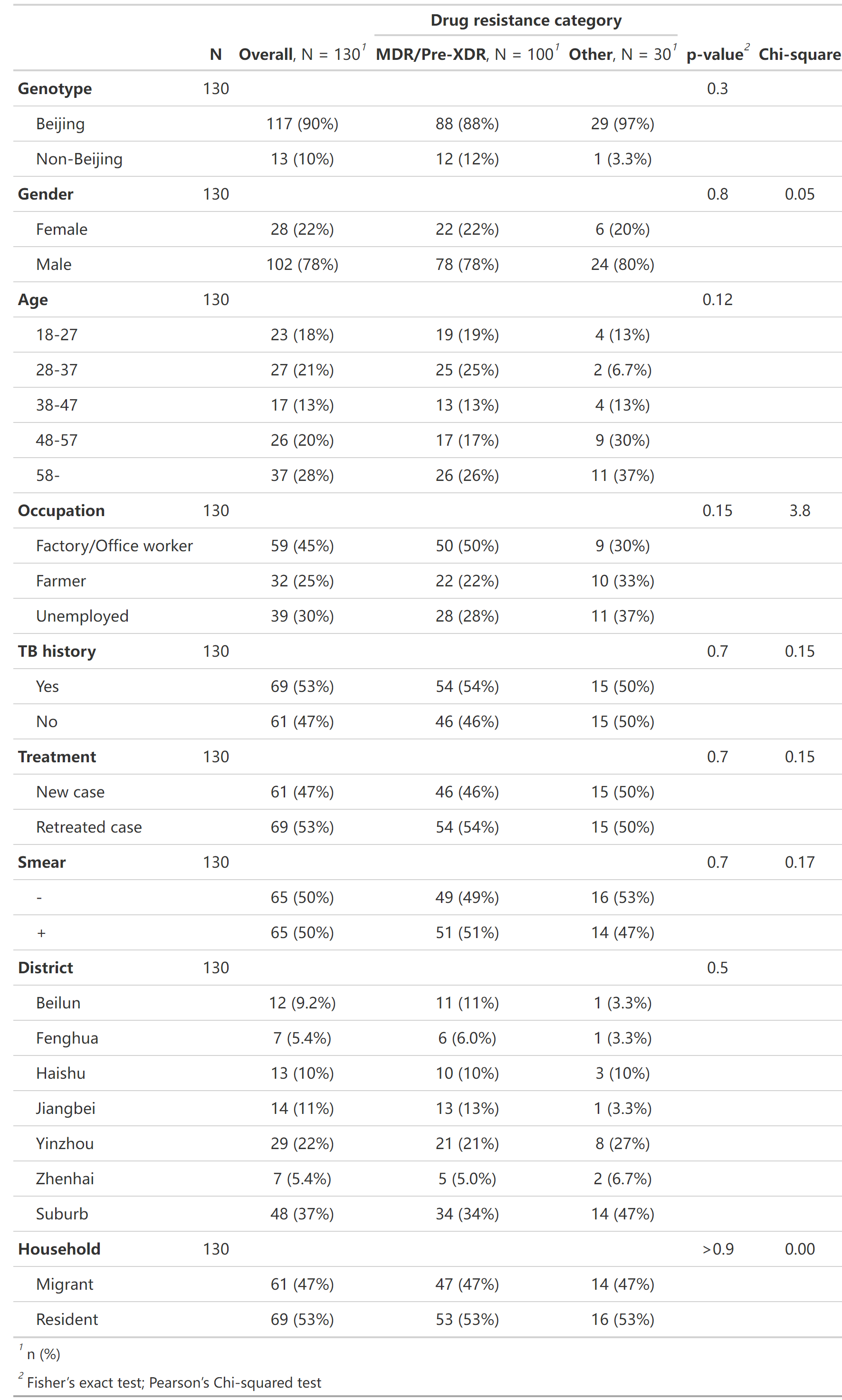

Supplement: Supplementary file 4 [file Table_2.docx]
