# Supplementary material for: Transmission dynamics of drug-resistant tuberculosis in Ningbo, China: an epidemiological and genomic analysis
Source: Front Cell Infect Microbiol. 2024 Feb 7;14:1327477. doi: 10.3389/fcimb.2024.1327477 (PMC10879548; doi:10.3389/fcimb.2024.1327477)
Supplement: Supplementary file 5 [file Table_3.docx]

**Supplementary Table S3. Whole-genome sequencing compared with phenotypic DST for detection of drug resistance**

| **Drug** | **Phenotypically resistant** | | **Phenotypically sensitive** | | **Consistency (%)** | **Sensitivity^a^ (%)** | **Specificity^a^ (%)** | **PPV^a^ (%)** | **NPV^a^ (%)** |
| --- | --- | --- | --- | --- | --- | --- | --- | --- | --- |
|  | **Genetically resistant** | **Genetically sensitive** | **Genetically resistant** | **Genetically sensitive** |  | **（95% CI, %）** | **（95% CI, %）** | **（95% CI, %）** | **（95% CI, %）** |
| INH | 96 | 4 | 4 | 26 | 93.85 | 96.00 (89.49-98.71) | 86.67 (68.36-95.64) | 96.00 (89.49-98.71) | 86.67 (68.36-95.64) |
| RIF | 124 | 0 | 4 | 2 | 96.92 | 100.00 (96.26-100.00) | - | 96.88 (91.71-98.99) | 100.00 (19.79-100.00) |
| SM | 80 | 2 | 3 | 45 | 96.15 | 97.56 (90.65-99.58) | 93.75 (81.79-98.37) | 96.39 (89.07-99.06) | 95.74 (84.27-99.26) |
| EMB | 43 | 2 | 27 | 58 | 77.69 | 95.56 (83.64-99.23) | 68.24 (57.13-77.68) | 61.43 (49.00-72.59) | 96.67 (87.45-99.42) |
| AM | 9 | 2 | 0 | 119 | 98.46 | 81.82 (47.76-96.79) | 100.00 (96.1-100.00) | 100.00 (62.88-100.00) | 98.35 (93.56-99.71) |
| CM | 6 | 2 | 3 | 119 | 96.15 | 75.00 (35.58-95.55) | 97.54 (92.44-99.36) | 66.67 (30.92-90.96) | 98.35 (93.56-99.71) |
| LVX | 48 | 2 | 1 | 79 | 97.69 | 96.00 (85.14-99.30) | 98.75 (92.27-99.93) | 97.96 (87.77-99.89) | 97.53 (90.54-99.57) |
| PAS | 8 | 10 | 3 | 109 | 90.00 | 44.44 (22.40-68.65) | 97.32 (91.79-99.31) | 72.73 (39.32-92.67) | 91.60 (84.71-95.67) |
| TH | 25 | 2 | 14 | 89 | 87.69 | 92.59 (74.25-98.71) | 86.41 (77.91-92.10) | 64.10 (47.15-78.32) | 97.80 (91.53-99.62) |

^a^CI was calculated for sensitivity, specificity, PPV, and NPV with the Wilson score confidence interval method.

INH, isoniazid; RIF, rifampicin; SM, streptomycin; EMB, ethambutol; AM, amikacin; CM, capreomycin; LVX, levofloxacin; PAS, para-aminosalicylic acid; TH, ethionamide; PPV, positive predictive value; NPV, negative predictive value; CI, confidence interval.
